# Total Blood Mercury Predicts Methylmercury Exposure in Fish and Shellfish Consumers

**DOI:** 10.1007/s12011-021-02968-9

**Published:** 2021-10-23

**Authors:** Ellen M. Wells, Leonid Kopylev, Rebecca Nachman, Elizabeth G. Radke, Johanna Congleton, Deborah Segal

**Affiliations:** 1 School of Health Sciences, Purdue University, West Lafayette, IN USA; 2 Department of Public Health, Purdue University, West Lafayette, IN USA; 3 Center for Public Health and Environmental Assessment, Office of Research and Development, US Environmental Protection Agency, 1200 Pennsylvania Ave, Washington DC, NW 20460 USA

**Keywords:** Mercury, Methylmercury, Reproductive age, NHANES, Biomarkers, Seafood

## Abstract

Many studies evaluating methylmercury (MeHg) toxicity rely on whole blood total mercury (THg) measurements to estimate MeHg exposure. However, whole blood THg includes other forms of mercury (Hg), such as inorganic Hg, which have different exposure sources and toxicological effects than MeHg. Therefore, estimating the whole blood MeHg/THg ratio is critical to predicting MeHg exposure and, subsequently, efforts to establish an exposure–response relationship for use in risk assessment. A large, representative dataset (National Health and Nutrition Examination Survey (NHANES) 2011–2016) was used to determine the whole blood MeHg/THg ratio among (a) self-reported fish and shellfish consumers, ≥ 15 years of age (the “full adult” population (*N* = 5268 training dataset; *N* = 2336 test dataset)) and (b) female fish and shellfish consumers, 15–44 years of age (the “women of reproductive age” population (*N* = 1285 training dataset; *N* = 560 test dataset)). Unadjusted and adjusted linear and spline models with direct measurements for both THg and MeHg were evaluated. The mean whole blood MeHg/THg ratio was 0.75 (95% confidence interval (CI): 0.74, 0.75). This ratio was significantly higher among those with higher THg concentrations. All models exhibited excellent fit (adjusted *R*^2^ from 0.957 to 0.982). Performance was slightly improved in spline versus linear models. For the full adult population and women of reproductive age, the unadjusted spline model predicted whole blood MeHg concentrations of 5.65 µg/L and 5.55 µg/L, respectively, when the THg concentration was 5.80 µg/L. These results suggest that whole blood THg is a good predictor of whole blood MeHg among fish and shellfish consumers.

**Supplementary Information:**

The online version contains supplementary material available at 10.1007/s12011-021-02968-9.

## Introduction

Mercury (Hg) is a naturally occurring heavy metal of great concern to public health. Ample research has demonstrated that exposure to various forms of Hg is associated with impaired neurodevelopment [1–3], nervous system effects in adults [4–6], cardiovascular diseases [7, 8], and renal toxicity [9, 10]. Globally, the World Health Organization (WHO) has designated Hg as one of the ten most dangerous chemicals to public health [11].

The chemical form of Hg affects its environmental fate and transport, sources of exposure, and toxicity. Health concerns have been associated with exposure to elemental Hg (Hg vapor) [5, 12], inorganic Hg [10, 13], and methylmercury (MeHg) [14, 15]. However, exposure to MeHg is a particular public health concern due to its well-documented neurotoxicity, particularly among children [14, 15]. Additionally, MeHg is the most common form of Hg to which humans are exposed [16], and a substantial proportion of the US population is likely exposed to MeHg at concentrations above recommended limits [17].

Consequently, regulatory agencies have developed guidelines specific to MeHg, including a reference dose by the US Environmental Protection Agency (US EPA) [18, 19]. The current reference dose, which is based on neurodevelopmental effects resulting from in utero exposure, is equivalent to a cord blood MeHg concentration of 5.80 µg/dL [19]. As it has been estimated that cord whole blood MeHg is 1.7 times higher than maternal whole blood [20], this would be equivalent to a maternal whole^1^ blood MeHg concentration of 3.40 µg/dL. However, the technology to measure MeHg and other specific forms of Hg directly has not been widely available and is expensive. Because MeHg was demonstrated to comprise approximately 90% of blood total mercury (THg) [16], the use of blood THg concentrations as a proxy for blood MeHg has been recommended [16, 21]. As a result, many research studies that evaluate the toxicity of MeHg have utilized measurements of blood THg, which includes MeHg as well as all forms of mercury, to estimate MeHg exposure.

More recent studies have suggested that the percentage of blood THg comprising MeHg may be highly variable as well as lower than previously estimated [22], with some results only reaching 61–63% [23]. This has led to concern that measuring THg, instead of MeHg, may result in exposure misclassification in which MeHg exposure is overestimated, leading to inexact estimations of its health effects [24, 25]. Thus, this poses a challenge for risk assessors, as it is unclear how to use the numerous studies that rely on measurements of blood THg in an exposure–response analysis for MeHg. Therefore, a model was developed to determine the relationship between THg and MeHg in blood. This was accomplished using data from the National Health and Nutrition Examination Survey (NHANES), which includes a large nationally representative population and direct measurements of both blood THg and MeHg. Although others have measured both THg and MeHg in blood, to the best of our knowledge, this manuscript presents the first model developed to predict blood MeHg based on blood THg measurements.

## Methods

We used data from NHANES, a cross-sectional survey conducted by the US Centers for Disease Control (CDC) of the non-institutionalized civilian US population in the 50 states and the District of Columbia. NHANES consists of a questionnaire and physical exam, during which blood samples are collected. At the time of analysis, directly measured THg and MeHg concentrations were available for NHANES data collected from 2011 to 2016. These were randomly divided into two datasets: one was used to create the prediction model, i.e., the “training” dataset, and the other was used to test the model, i.e., the “test” dataset. NHANES participants sign informed consent documentation prior to participation; NHANES operates under an approved protocol from the National Center for Health Statistics (NCHS) Ethics Review Board. More details about NHANES are available online at https://www.cdc.gov/nchs/nhanes/index.htm.

A study participant flowchart is presented in Supplemental Figure S1. There were *N* = 29,902 participants in NHANES 2011–2016. *N* = 11,953 participants were excluded because they did not have THg and/or MeHg blood measurements. Of note is that, in 2011–2012, NHANES measured THg and MeHg for the entire population providing a blood sample, but starting in 2013, only a random 50% sample of the full population who provided blood had their samples analyzed for Hg. Participants with missing data on other model covariates (income, *N* = 1409; body mass index (BMI), *N* = 586; self-reported fish consumption, *N* = 178) were also excluded. As this analysis is heavily reliant on laboratory measurements of blood Hg, we excluded groups with a high rate of blood Hg concentrations below the detection limit: younger participants (< 15 years old; 33.5% of THg was < LOD) and non-fish or shellfish consumers (31.9% of THg was < LOD). *N* = 5323 participants were excluded because they were less than 15 years old, and *N* = 2,867 participants were excluded because they did not report eating fish or shellfish within the past 30 days. This left a total of *N* = 7604 for analyses, referred to as the “full adult” population. In addition, as much of the existing epidemiologic studies on Hg focuses on prenatal exposures, we were specifically interested in women of reproductive age. Thus, we also created models within the subset of female fish and shellfish consumers who were of reproductive age (15 to 44 years, *N* = 1845), referred to as the “women of reproductive age” population. Two-thirds of this population was randomly selected to be in the training dataset (*N* = 5268 full adult, *N* = 1285 women of reproductive age) and the remainder were included in the test dataset (*N* = 2336 full adult, *N* = 560 women of reproductive age).

Blood samples were collected by trained phlebotomists using metal-free containers. Samples were frozen (− 30° C) until shipment to the US CDC. They were transferred to the US CDC Division of Laboratory Sciences (Atlanta, Georgia) within the National Center for Environmental Health for Hg determination. All quality assurance and quality control protocols for Hg assessment meet the 1988 Clinical Laboratory Improvement Act mandates [26, 27]. Other than the change in the limit of detection for THg (described below), the laboratory procedures did not change over time.

Blood THg was determined using quadrupole inductively coupled plasma mass spectrometry (ICP-MS) (ELAN DRC II; PerkinElmer, Norwalk, CT, USA). This method detects the mass-to-charge ratio for each ion in the sample, which is used to determine the element and its concentration. The limit of detection (LOD) for blood THg was 0.16 µg/L in 2011–2012 and 0.28 in 2013–2016. To ensure that this did not cause any bias in our analysis, a uniform LOD (0.28) was applied to all THg samples; this affected 163/5268 measurements, or 3.1% of the full population training dataset. Values < LOD were replaced with LOD/√(2) for analyses. In the training dataset, there were 407/5268 (7.7%) values < LOD (full adult) and 121/1825 (9.4%) values < LOD (women of reproductive age). Corresponding values for the test dataset were 172/2336 (7.4%) (full adult) and 58/560 (10.4%) (women of reproductive age).

MeHg concentration was determined using a triple spike isotope dilution (TSID) method; gas chromatography (GC) (Clarus 500; PerkinElmer, Norwalk, CT, USA) was used to separate Hg forms. This was followed by inductively coupled plasma dynamic reaction cell mass spectrometry (ICP-DRC-MS) (NexION 300D; PerkinElmer, Norwalk, CT, USA) for quantification. Hg forms in blood were measured using stannous chloride as a reductant. This method has a MeHg LOD of 0.12 µg/L; this did not change over the time period included in this analysis. Similar to THg, values < LOD were replaced with LOD/√(2). In the training dataset, there were 351/5268 (6.7%) values < LOD for MeHg (full adult) and 104/1285 (8.1%) values < LOD (women of reproductive age). Corresponding values for the test dataset were 129/2336 (5.5%) (full adult) and 37/560 (6.6%) (women of reproductive age).

Demographic data (age, sex, fish and shellfish consumption, race/ethnicity, income) were obtained via questionnaire. Categories used for race/ethnicity were non-Hispanic white, non-Hispanic black, Hispanic (indicated as “Mexican–American” or “Other Hispanic”), Asian, and mixed race/other. Income was defined as annual household income. Weight and height were collected at the physical examination. BMI was calculated as weight (kg) / (height (m))^2^ and classified as normal or underweight (BMI < 25), overweight (BMI 25 to 29.9), and obese (BMI ≥ 30).

Stata 13.0 (College Station, TX, USA) was used for statistical analyses; a *p* value of 0.05 was considered to be statistically significant, using Pearson or Wald Chi-square tests as appropriate. The training dataset was used to create a model which would predict MeHg concentrations. NHANES is designed to obtain a representative sample of the USA; the use of appropriate survey weights and analytic methods is needed to obtain statistical results that reflect this representative sampling. However, as the goal for this analysis is not to describe the prevalence of MeHg within the US population and not all model development statistics are readily applied to survey data, survey weights and analytic methods were not used in this analysis. This could affect the variance of some variables; it also means that the population included in the analysis does not reflect a representative sample of the USA.

We explored the relationship between MeHg and THg blood concentrations. Both were approximately lognormally distributed; thus, central tendencies are reported using geometric means and log scales are used in some figures. Several unadjusted models with blood THg as the independent variable and blood MeHg as the dependent variable were created to explore model fit, including linear, linear with natural-log transformations for MeHg and/or THg, spline, and cubic spline models. We evaluated model fit using model *R*^2^, mean squared error (MSE), Akaike’s Information Criterion (AIC), Bayesian Information Criterion (BIC), residuals, leverage, residual versus fitted (RVF) plots, and leverage versus squared residual (LVR2) plots. Model covariates were selected based on preliminary evaluation of their associations with blood MeHg and THg; additionally, these are commonly used covariates which have been reported to be associated with MeHg and THg in other studies. Specifically, associations of the MeHg/THg ratio have been reported with age [28, 29], sex [22], race/ethnicity [28], and fish and shellfish consumption [29]. Model covariates included age (continuous), sex (binary), race/ethnicity (categorical), income (categorical), and BMI (categorical). Model fit statistics described above were used to evaluate model fit. These models were also used to predict blood MeHg concentrations using the test dataset.

## Results

Unadjusted models were created for linear, linear with natural-log transformations for MeHg and/or THg, spline, and cubic spline models. The natural-log transformed models had the poorest fit and were therefore not considered in detailed analyses (data not shown). The spline models, including the cubic spline, were roughly similar, so the best fitting spline model, with a knot at 1 µg/L THg, was used in further analyses along with the linear model.

Demographic characteristics are shown in Table 1. Across both the training and test datasets, the mean age is 46.3 (95% confidence interval (CI): 45.9, 46.7) (full adult) and 29.3 (95% CI: 28.9, 29.7) (women of reproductive age). A majority of participants are non-Hispanic white, have household incomes greater than $45,000, and are classified as overweight or obese. Women of reproductive age in the training dataset had a statistically significant higher income than women in the test datasets; however, this did not appear to substantially influence results.

**Table 1:** Population characteristics among fish and shellfish consumers, NHANES 2011–2016

	Full adult population			Women of reproductive age		
	Training dataset	Test dataset	*p*	Training dataset	Test dataset	*p*
*N*	5268	2336		1285	560	
Age						
15–29	1272 (24.2)	568 (24.3)		644 (50.1)	283 (50.5)	
30–44	1241 (23.6)	534 (22.9)		641 (49.9)	277 (49.5)	0.869
45–59	1220 (23.2)	535 (22.9)		–	–	
60 +	1535 (29.1)	699 (29.9)	0.863	–	–	
Sex						
Female	2704 (51.3)	1177 (50.4)		–	–	
Male	2564 (48.7)	1159 (49.6)	0.448	–	–	
Race/ethnicity						
NH white	1965 (37.3)	922 (39.5)		377 (29.3)	176 (31.4)	
NH black	1300 (24.7)	537 (23.0)		337 (26.2)	132 (23.6)	
Hispanic	1176 (22.3)	538 (23.0)		341 (26.5)	152 (27.1)	
NH Asian	637 (12.1)	273 (11.7)		176 (13.7)	82 (14.6)	
Multiracial/other	190 (3.6)	66 (2.8)	0.115	54 (4.2)	18 (3.2)	0.570
Household income						
< $20,000	1060 (20.1)	518 (22.2)		**229 (17.8)**	**132 (23.6)**	
$20,000 to $44,999	1587 (30.1)	678 (29.0)		**397 (30.9)**	**163 (29.1)**	
$45,000 to $74,999	1029 (19.5)	441 (18.9)		**268 (20.9)**	**105 (18.8)**	
≥ $75,000	1592 (30.2)	699 (29.9)	0.226	**391 (30.4)**	**160 (28.6)**	**0.040**
Body mass index						
< 25 kg/m^2^	1732 (32.9)	747 (32.0)		537 (41.8)	226 (40.4)	
25 to 29.9 kg/m^2^	1619 (30.7)	739 (31.6)		299 (23.3)	137 (24.5)	
≥ 30 kg/m^2^	1917 (36.4)	850 (36.4)	0.661	449 (34.9)	197 (35.2)	0.804
No. of seafood meals/30 days						
1 to 2 meals	1736 (33.0)	760 (32.5)		472 (36.7)	198 (35.4)	
3 meals	605 (11.5)	309 (13.2)		161 (12.5)	80 (14.3)	
4 to 7 meals	1527 (29.0)	670 (28.7)		325 (25.3)	149 (26.6)	
≥ 8 meals	1400 (26.6)	597 (25.6)	0.182	327 (25.5)	133 (23.8)	0.607

Full: ≥ 15 years old; Reproductive: women 15 to 44 years old. *NHANES*, National Health and Nutrition Examination Survey; *NH*, non-Hispanic. Values are percent (95% confidence interval). Pearson’s chi-square test is used to compare training versus test datasets; bold type indicates *p* < 0.05

**Fig. 1:**
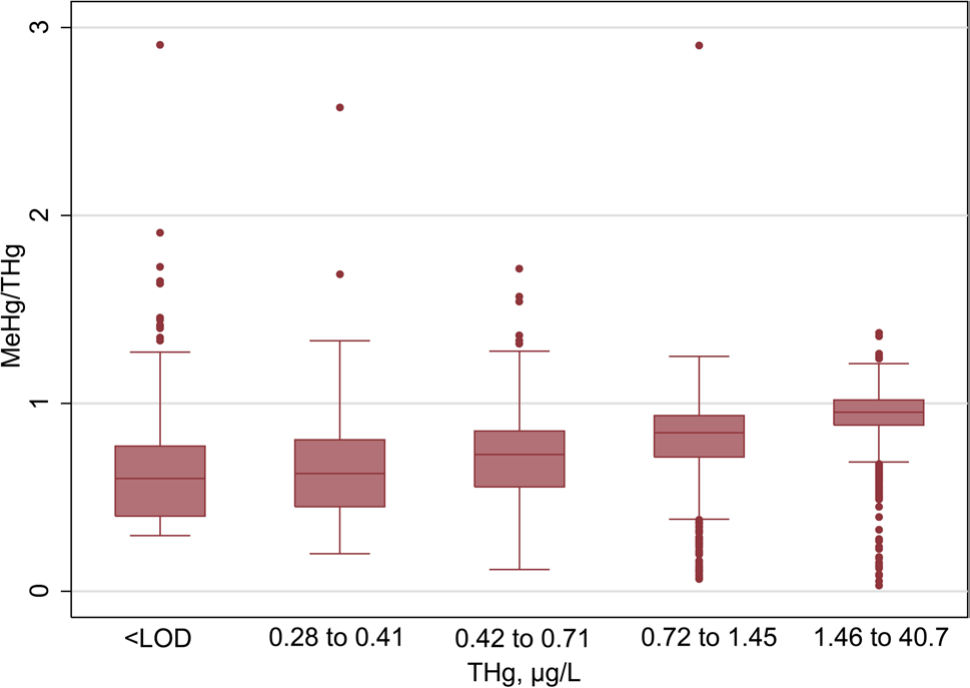
Boxplots displaying the distribution of methylmercury/total mercury (*y*-axis) by quintile of total mercury (*x*-axis) among fish and shellfish consumers at least 15 years of age (full adult population) from the training dataset, *N* = 5268

Average blood Hg concentrations and the MeHg/THg ratio are presented in Table 2. Across both the training and test datasets, the geometric mean (95% CI) was 0.99 µg/L (0.97, 1.01) for THg and 0.74 µg/L (0.72, 0.76) for MeHg; mean (95% CI) MeHg/THg was 0.80 (0.79, 0.80). There were no statistically significant differences in these values in the training versus test datasets. The relationship between MeHg and THg was visually displayed using nonparametric lowess plots (Supplementary Information, Figure S2) and box plots of the MeHg/THg ratio by quartile of THg (Fig. 1). The MeHg/THg ratio was significantly higher among those with higher THg concentrations: the average MeHg/THg ratio was 0.65 for those in the lowest quintile of THg but was 0.94 for those in the highest quintile of THg (Fig. 1).

**Table 2:** Whole blood mercury concentrations, fish and shellfish consumers, NHANES 2011–2016

	Full adult population			Women of reproductive age		
	Training dataset	Test dataset	*p*	Training dataset	Test dataset	*p*
*N*, all	5268	2336		1285	560	
THg, µg/L	0.99 (0.97, 1.02)	0.99 (0.95, 1.03)	0.975	0.84 (0.80, 0.88)	0.83 (0.77, 0.90)	0.870
MeHg, µg/L	0.74 (0.71, 0.76)	0.74 (0.71, 0.78)	0.922	0.59 (0.56, 0.63)	0.61 (0.55, 0.67)	0.740
MeHg/THg	0.75 (0.74, 0.75)	0.75 (0.74, 0.76)	0.495	0.71 (0.69, 0.73)	0.73 (0.70, 0.75)	0.292

Full: ≥ 15 years old; Reproductive: women 15 to 44 years old. *NHANES*, National Health and Nutrition Examination Survey; *THg*, total mercury; *MeHg*, methylmercury. Values for THg and MeHg are geometric mean (95% confidence interval); values for MeHg/THg are mean (95% confidence interval). *p* values are a comparison of training versus test datasets using a Wald test

Average blood Hg concentrations and the MeHg/THg ratio stratified by demographic characteristics are presented in Table 3. THg and MeHg blood concentrations were significantly higher among those of older age, men, non-Hispanic Asians, non-Hispanic blacks (full adult population only), participants with a higher household income, and participants with a lower BMI. The MeHg/THg ratio was also higher among these groups.

**Table 3:** Total and methylmercury concentrations by selected covariates, fish and shellfish consumers, NHANES 2011–2016

Variable	Full adult population (*n* = 7604)			Women of reproductive age (*n* = 1845)		
	THg	MeHg	MeHg/THg	THg	MeHg	MeHg/THg
Age						
15–29 ^a^	0.76 (0.73, 0.79)	0.56 (0.53, 0.59)	0.79 (0.78, 0.81)	0.75 (0.71, 0.80)	0.54 (0.50, 0.58)	0.78 (0.76, 0.80)
30–44	**0.98 (0.94, 1.03)**	**0.73 (0.69, 0.77)**	0.79 (0.78, 0.80)	**0.93 (0.88, 0.99)**	**0.66 (0.61, 0.71)**	0.76 (0.75, 0.78)
45–59	**1.21 (1.15, 1.26)**	**0.92 (0.86, 0.97)**	0.81 (0.80, 0.82)	–	–	–
60 +	**1.06 (1.02, 1.10)**	**0.79 (0.75, 0.83)**	0.80 (0.79, 0.81)	–	–	–
Sex						
Female ^a^	0.96 (0.93, 0.99)	0.69 (0.67, 0.72)	0.78 (0.77, 0.79)	–	–	–
Male	**1.02 (0.99, 1.06)**	**0.79 (0.76, 0.82)**	**0.82 (0.81, 0.82)**	–	–	–
Race/ethnicity						
NH white ^a^	0.86 (0.83, 0.89)	0.61 (0.58, 0.64)	0.77 (0.76, 0.78)	0.73 (0.67, 0.78)	0.51 (0.46, 0.56)	0.76 (0.74, 0.78)
NH black	**0.92 (0.88, 0.95)**	**0.72 (0.69, 0.75)**	**0.82 (0.81, 0.83)**	0.75 (0.69, 0.81)	0.54 (0.49, 0.59)	0.77 (0.75, 0.79)
Hispanic	0.82 (0.79, 0.86)	*0.57 (0.55, 0.60)*	*0.75 (0.74, 0.77)*	0.69 (0.65, 0.74)	*0.45 (0.42, 0.50)*	**0.72 (0.70, 0.74)**
NH Asian	**2.61 (2.46, 2.78)**	**2.36 (2.20, 2.54)**	**0.93 (0.92, 0.94)**	**2.01 (1.79, 2.25)**	**1.73 (1.51, 1.99)**	**0.90 (0.87, 0.92)**
Multiracial/other	0.92 (0.81, 1.04)	0.68 (0.58, 0.79)	0.79 (0.76, 0.81)	0.80 (0.64, 1.02)	0.56 (0.41, 0.75)	0.75 (0.69, 0.80)
Household income						
< $20,000 ^a^	0.81 (0.77, 0.84)	0.57 (0.54, 0.61)	0.77 (0.76, 0.79)	0.71 (0.65, 0.77)	0.48 (0.43, 0.54)	0.75 (0.72, 0.78)
$20,000 to $44,999	0.83 (0.80, 0.86)	0.60 (0.57, 0.63)	0.77 (0.76, 0.78)	0.70 (0.65, 0.74)	0.47 (0.43, 0.52)	0.74 (0.72, 0.76)
$45,000 to $74,999	**1.00 (0.95, 1.05)**	**0.74 (0.69, 0.78)**	*0.79 (0.78, 0.80)*	**0.81 (0.74, 0.88)**	**0.57 (0.50, 0.64)**	0.76 (0.74, 0.79)
≥ $75,000	**1.34 (1.29, 1.40)**	**1.08 (1.03, 1.13)**	**0.84 (0.83, 0.85)**	**1.16 (1.06, 1.26)**	**0.90 (0.82, 1.00)**	**0.83 (0.81, 0.85)**
BMI						
Under/normal weight ^a^	1.12 (1.07, 1.16)	0.86 (0.82, 0.91)	0.82 (0.81, 0.83)	0.97 (0.90, 1.04)	0.72 (0.65, 0.78)	0.80 (0.78, 0.81)
Overweight	**1.03 (0.99, 1.07)**	**0.77 (0.73, 0.81)**	**0.80 (0.79, 0.81)**	**0.84 (0.77, 0.91)**	**0.61 (0.55, 0.67)**	0.78 (0.76, 0.80)
Obese	**0.86 (0.84, 0.89)**	**0.62 (0.59, 0.64)**	**0.77 (0.76, 0.78)**	**0.70 (0.66, 0.75)**	**0.48 (0.44, 0.52)**	**0.74 (0.72, 0.76)**

Full: ≥ 15 years old; Reproductive: women 15 to 44 years old. Data include both training and test datasets. Statistical tests were Wald tests to determine whether there was a significant difference in mercury concentration compared to the referent group. Bold type: Wald test *p* < 0.05. Italic type: *p* < 0.10. ^a^Referent

Regression model results using the training dataset to predict blood MeHg are presented in Tables S1 and S2. In the unadjusted linear model among the full adult population, the *β* coefficient for THg was 1.01 (95% CI: 1.00, 1.01). In unadjusted spline models among the full adult population, the *β* coefficient for THg values ≤ 1 µg/L was 0.88 (95% CI: 0.85, 0.91), and *β* for THg > 1 µg/L was 1.01 (95% CI: 1.01, 1.02). *β* coefficients for THg were not substantially affected by the population or adjustment for additional covariates; all THg coefficients were statistically significant. In adjusted models, other coefficients which were statistically significant included age (women of reproductive age only), sex (full adult only), Hispanic (vs. non-Hispanic white, women of reproductive age only), Asian (vs. non-Hispanic white, full adult only) and non-Hispanic black (vs. non-Hispanic white, full adult only), income (women of reproductive age only), and obese (vs. normal weight, full adult only). An increase in blood THg at low concentrations (≤ 1 µg/L) was associated with a significantly smaller increase in estimated blood MeHg (*β*: 0.88, 95% CI: 0.85, 0.91) compared to an increase in THg at higher concentrations (> 1 µg/L) (*β*: 1.01, 95% CI: 1.01, 1.02).

Indicators of model fit for the training dataset are shown in Table 4 and Figures S3 to S6 (Supplementary Information). These were very similar across the different models, and overall indicated a very good model fit. *R*^2^ values for models among the full adult population, rounded to three decimal places, were 0.982, and ranged from 0.957 to 0.959 for the women of reproductive age population. Median model residuals ranged from 0.02 µg/L (interquartile range (IQR): − 0.07, 0.12) in the unadjusted spline model for the women of reproductive age population to 0.04 µg/L (IQR: − 0.09, 0.12) in the unadjusted linear model for the full adult population.

**Table 4:** Model performance indicators, fish and shellfish consumers, training dataset

Model	Adj. *R*^2^	Root MSE	AIC	BIC	Predicted MeHg ^a^	Residual ^a^	Leverage × 1000 ^a^
Full adult population^b^							
Linear							
Unadjusted	0.982	0.318	2883	2896	0.74 (0.34, 1.70)	0.04 (− 0.09, 0.12)	0.23 (0.20, 0.25)
Adjusted	0.982	0.316	2837	2922	0.74 (0.33, 1.70)	0.03 (− 0.08, 0.12)	2.15 (1.90 2.47)
Spline							
Unadjusted	0.982	0.316	2826	2845	0.71 (0.36, 1.67)	0.02 (− 0.09, 0.12)	0.38 (0.33, 0.48)
Adjusted	0.982	0.314	2768	2860	0.71 (0.35, 1.67)	0.02 (− 0.09, 0.11)	2.33 (2.06, 2.70)
Women of reproductive age^c^							
Linear							
Unadjusted	0.957	0.347	928	938	0.60 (0.29, 1.34)	0.03 (− 0.07, 0.11)	0.97 (0.85, 1.09)
Adjusted	0.959	0.342	904	965	0.60 (0.27, 1.32)	0.03 (− 0.08, 0.12)	8.15 (7.16, 9.54)
Spline							
Unadjusted	0.958	0.346	922	938	0.59 (0.31, 1.31)	0.02 (− 0.07, 0.12)	1.72 (1.27, 2.16)
Adjusted	0.959	0.341	897	964	0.59 (0.29, 1.30)	0.02 (− 0.08, 0.12)	8.98 (7.87, 10.36)

*MeHg*, methylmercury; *MSE*, mean squared error; *AIC*, Akaike’s information criterion; *BIC*, Bayesian Information Criterion

^a^Median (interquartile range) in μg/L

^b^Full: ≥ 15 years old, *N* = 5268. Median (interquartile range) for measured MeHg is 0.71 (0.32, 1.68)

^c^Reproductive: women 15 to 44 years old, *N* = 1285. Median (interquartile range) for measured MeHg is 0.55 (0.27, 1.32)

The linear THg and spline THg models were then used to predict MeHg using the test dataset; model fit statistics are presented in Table 5 and Figures S7 to S10 (Supplementary Information). Overall, these also suggested excellent model fit. Median model residuals ranged from 0.02 (interquartile range (IQR): − 0.09, 0.12) in the spline models among the full adult population to 0.03 (IQR: − 0.09, 0.12) in the linear model among the full adult population.

**Table 5:** Model performance indicators, fish and shellfish consumers, test dataset

Population and model	Measured MeHg^a^	Predicted MeHg^a^	Residual^a^	Leverage × 1000^a^
Full adult population (*n* = 2336)				
Linear				
Unadjusted	0.70 (0.33, 1.61)	0.72 (0.34, 1.63)	0.03 (− 0.09, 0.12)	0.23 (0.20, 0.25)
Adjusted	0.70 (0.33, 1.61)	0.72 (0.33, 1.64)	0.03 (− 0.09, 0.12)	2.13 (1.90, 2.45)
Spline				
Unadjusted	0.70 (0.33, 1.61)	0.69 (0.36, 1.60)	0.02 (− 0.09, 0.12)	0.39 (0.33, 0.47)
Adjusted	0.70 (0.33, 1.61)	0.70 (0.36, 1.61)	0.02 (− 0.09, 0.12)	2.33 (2.05, 2.69)
Women of reproductive age (*N* = 560)				
Linear				
Unadjusted	0.58 (0.27, 1.29)	0.56 (0.27, 1.31)	0.03 (− 0.07, 0.11)	0.96 (0.85, 1.09)
Adjusted	0.58 (0.27, 1.29)	0.59 (0.26, 1.29)	0.02 (− 0.07, 0.13)	8.15 (7.16, 9.51)
Spline				
Unadjusted	0.58 (0.27, 1.29)	0.55 (0.29, 1.28)	0.03 (− 0.07, 0.12)	1.69 (1.27, 2.15)
Adjusted	0.58 (0.27, 1.29)	0.57 (0.29, 1.26)	0.02 (− 0.07, 0.12)	8.87 (7.84, 10.34)

Full: ≥ 15 years old; Reproductive: women 15 to 44 years old. ^a^Median (interquartile range) in µg/L

Estimated MeHg for selected values for THg using this unadjusted spline model are presented in Table S3 (Supplementary Information). For a THg blood concentration of 3.40 µg/L, the predicted blood MeHg concentration would be 3.22 µg/L among the full adult population and 3.18 µg/L among women of reproductive age. Corresponding values for a blood THg concentration of 5.80 µg/L are blood MeHg concentrations of 5.65 µg/L and 5.55 µg/L, respectively, for the full adult population and the women of reproductive age population.

## Discussion

This analysis used a representative sample of the adult population of fish and shellfish consumers and a subset of women of reproductive ages from the USA to develop and test a model which would predict blood MeHg concentrations using blood THg concentrations. After testing several models, we selected the unadjusted spline model for prediction of MeHg: its performance is excellent, and due to its relative simplicity, it can be applied even when a limited amount of data from the original study is available.

Although we are not aware of other models that estimate blood MeHg based on blood THg, several investigators have reported on a key parameter influencing this model: the MeHg/THg ratio. This analysis of the training dataset found that the average blood MeHg/THg ratio was 0.75 (Table 2). This is similar to reported values: 0.69 to 0.85 from other analyses of NHANES data [28, 30]; 0.63 from pregnant women in North Carolina [23]; 0.52 to 0.88 from populations in Europe [24, 31–34]; 0.86 from pregnant women in Suriname [35]; 0.72 to 0.93 from populations in Asia [22, 29, 36]; and 0.91 among newcomers to Canada [37]. Although the exact reason for the variation across populations is not known, it is possible this is related to differences in demographics or diet. Across both datasets, significant associations were observed between the MeHg/THg ratio and sex, race/ethnicity, income, BMI, and fish and shellfish consumption (Table 3).

In this analysis, the MeHg/THg ratio was higher among those with higher THg concentrations. This positive correlation of the MeHg/THg ratio with THg concentration has also been observed in several prior studies [23, 24, 29, 38]. Interestingly, two studies have also reported negative correlations [22, 23]. However, these might be explained by differences in study design (measurement in late pregnancy) [23] or population (substantially higher THg concentrations) [22]. Among those with lower THg exposure, there may be a higher proportion of elemental or inorganic Hg from sources such as dental amalgams or some foods [24, 39].

This analysis has a few limitations. First, the detection limit for THg in several NHANES cycles was high (0.28 µg/L). Due to this, some groups with lower THg exposure (children and those who do not consume fish or shellfish) were not included in this analysis. Additionally, estimates for blood MeHg for those with lower THg concentrations within our model may not be as precise as the estimates for those with higher THg exposure. Second, as we did not use survey weights in our analysis, our results cannot be assumed to reflect a representative sample of the US population. However, as the dataset was large, we feel that results are still robust.

There are also several strengths of this analysis. These results are likely to be of great use to risk assessors who need to synthesize data from studies on health risks related to blood THg instead of blood MeHg. They are also consistent with the prior literature which compared blood THg to blood MeHg concentrations. Additionally, we present results specific to women of reproductive age, the demographic group commonly included in studies of the health effects of MeHg exposure.

## Conclusions

This manuscript describes the development of the first model, of which we are aware, that predicts whole blood MeHg based on whole blood THg. For studies evaluating MeHg toxicity that rely only on blood THg, this model can be used to convert blood THg concentrations to blood MeHg concentrations, and, therefore, to provide accurate estimates of exposure to MeHg.

## Supplementary Information

Below is the link to the electronic supplementary material.

Supplementary file1 (PDF 4511 KB)

## Data Availability

National Health and Nutrition Examination Survey (NHANES) data are available from the US Centers for Disease Control and Prevention at https://www.cdc.gov/nchs/nhanes/. Stata code and additional quality assurance details used in this analysis are available from the US EPA’s ScienceHub website at 10.23719/1520665.

## Abbreviations

AIC: Akaike’s Information Criterion
BIC: Bayesian Information Criterion
BMI: Body mass index
ICP-DRS-MS: Inductively coupled plasma dynamic reaction cell mass spectrometry
IC-PMS: Inductively coupled plasma mass spectrometry
IQR: Interquartile range
LOD: Limit of detection
LVR2: Leverage versus squared residual
MeHg: Methylmercury
MSE: Mean squared error
NCHS: National Center for Health Statistics
NHANES: National Health and Nutrition Examination Survey
RVF: Residual versus fitted
THg: Total mercury
TSID: Triple spike isotope dilution
US CDC: United States Centers for Disease Control and Prevention
US EPA: United States Environmental Protection Agency
USA: United States
WHO: World Health Organization

## Footnotes

1 In the rest of the article, whole blood will be referred to as blood.

## Code Availability

Upon publication of the manuscript, the code will be available at https://catalog.data.gov/dataset/epa-sciencehub.
