# Supplementary material for: Total Blood Mercury Predicts Methylmercury Exposure in Fish and Shellfish Consumers
Source: Biol Trace Elem Res. 2021 Oct 23;200(8):3867–75. doi: 10.1007/s12011-021-02968-9 (PMC9200869; doi:10.1007/s12011-021-02968-9)
Supplement: Supplementary file 1 — Supplementary file1 (PDF 4511 KB) [file 12011_2021_2968_MOESM1_ESM.pdf]

Supplementary Material

Total Blood Mercury Predicts Methylmercury Exposure In Fish and Shellfish Consumers

Ellen M. Wells,[1,2] Leonid Kopylev,[3] Rebecca Nachman,[3] Elizabeth G Radke,[3] Johanna Congleton,[3] Deborah Segal[3]

1. School of Health Sciences, Purdue University, West Lafayette, Indiana, USA
2. Department of Public Health, Purdue University, West Lafayette, Indiana, USA
3. Center for Public Health and Environmental Assessment, Office of Research and Development, US Environmental Protection Agency, Washington DC, USA

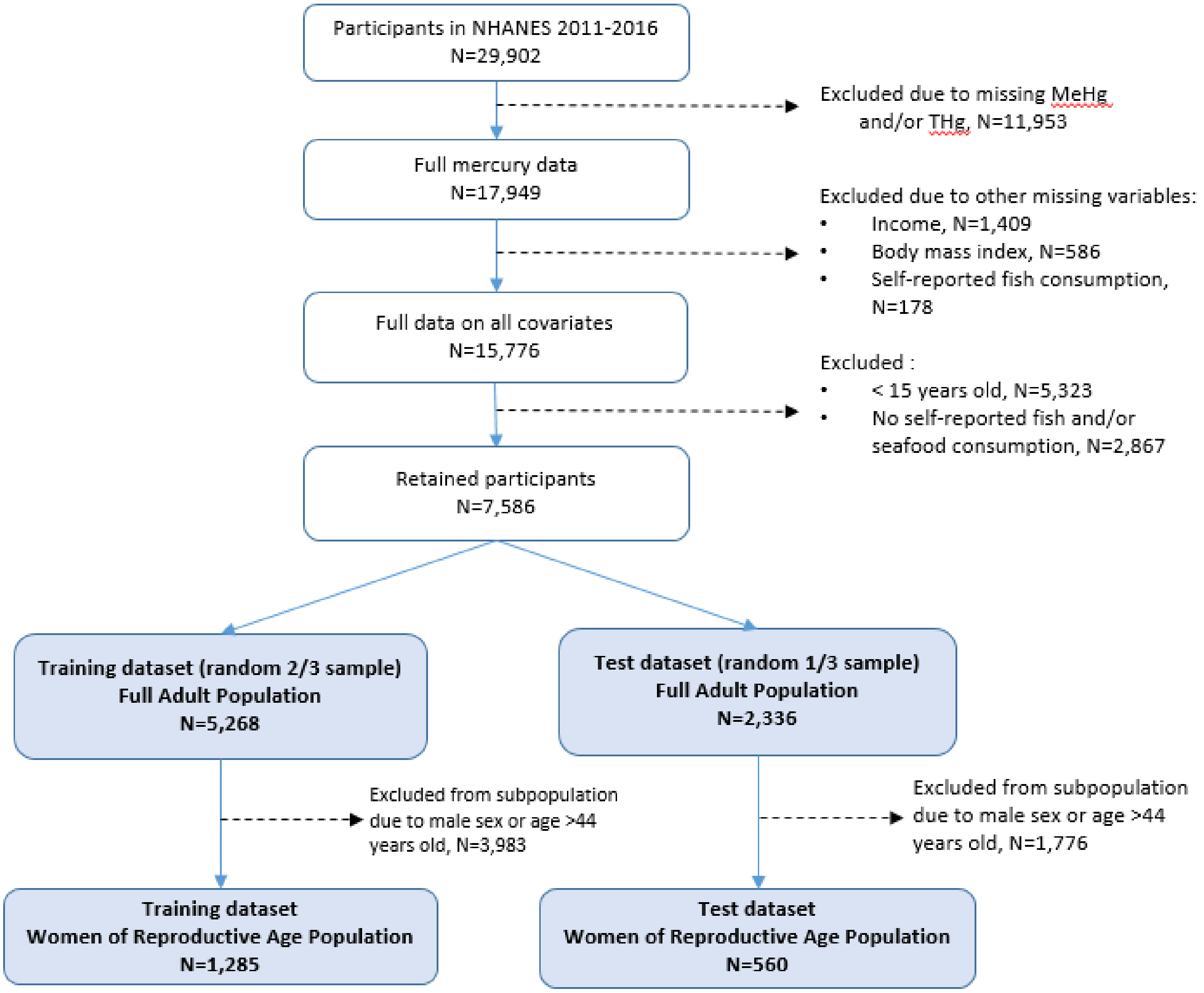

Figure S1: Flowchart of study participants. NHANES=National Health and Nutrition Examination Survey.

Table S1: Model results, full adult population, N=5268, training dataset

	Linear THg models		Spine THg models	
	Unadjusted	Adjusted	Unadjusted	Adjusted
Constant	**-0.17 (-0.18, -0.16)**	**-0.15 (-0.19, -0.12)**	**-0.08 (-0.11, -0.06)**	**-0.08 (-0.12, -0.04)**
THg (continuous)	**1.01 (1.00, 1.01)**	**1.00 (1.00, 1.01)**		
THg ≤ 1 µg/L			**0.88 (0.85, 0.91)**	**0.86 (0.83, 0.90)**
THg > 1 µg/L			**1.01 (1.01, 1.02)**	**1.01 (1.01, 1.01)**
Age (per 10 years)		-0.003 (-0.008 0.002)		-0.0005 (-0.005, 0.004)
Sex				
Female[a]				
Male		**0.03 (0.01, 0.05)**		**0.03 (0.01, 0.04)**
Race/ethnicity				
NH White [a]				
NH Black		**0.03 (0.004, 0.05)**		**0.03 (0.01, 0.06)**
Hispanic		-0.02 (-0.04, 0.01)		-0.01 (-0.04, 0.01)
NH Asian		**0.08 (0.05, 0.11)**		**0.09 (0.06, 0.12)**
Multiracial/other		0.03 (-0.02, 0.07)		0.03 (-0.02, 0.08)
Household income				
<$20,000 [a]				
$20,000 to $44,999		-0.01 (-0.03, 0.01)		-0.01 (-0.03, 0.01)
$45,000 to $74,999		-0.02 (-0.05, 0.01)		-0.02 (-0.04, 0.01)
≥$75,000		0.003 (-0.02, 0.03)		0.01 (-0.01, 0.04)
BMI				
BMI < 25 kg/m^2 [a]				
BMI 25 to 29.9 kg/m^2		-0.01 (-0.04, 0.01)		-0.01 (-0.03, 0.01)
BMI > 30 kg/m^2		**-0.03 (-0.05, -0.003)**		**-0.02, (-0.05, -0.002)**

Values are β coefficient (95% confidence interval)**. Bold type indicates p<0.05**; *italic type indicates p<0.10* for a Wald test comparing the value to the referent or for an increase of one unit. NHANES = National Health and Nutrition Examination Survey; THg = total mercury; NH = Non-Hispanic; BMI = Body mass index. a. Referent

Table S2: Model results, women of reproductive age population, N=1285, training dataset

Model	Linear		Spline	
**Adjustment level**	**Unadjusted**	**Adjusted**	**Unadjusted**	**Adjusted**
Constant	**-0.15 (-0.17, -0.12)**	-0.01 (-0.10, 0.07)	**-0.08 (-0.13, -0.03)**	0.04 (-0.05, 0.13)
THg (continuous)	**0.98 (0.97, 0.99)**	**0.98 (0.96, 0.99)**		
THg ≤ 1 µg/L			**0.88 (0.81, 0.95)**	**0.87 (0.80, 0.94)**
THg >1 µg/L			**0.99 (0.98, 1.01)**	**0.99 (0.97, 1.00)**
Age (per 10 years)		**-0.04 (-0.06, -0.02)**		**-0.04 -(0.06, -0.02)**
Race/ethnicity				
NH White				
NH Black		*-0.04 (-0.10, 0.01)*		-0.04 (-0.09, 0.01)
Hispanic		**-0.10 (-0.15, -0.04)**		**-0.09 (-0.14, -0.04)**
NH Asian		0.02 (-0.08, 0.08)		0.03 (-0.04, 0.09)
Multiracial/other		0.004 (-0.09, 0.10)		0.003 (-0.10, 0.10)
Household income				
<$20,000[a]				
$20,000 to $44,999		-0.001 (-0.06, 0.06)		-0.004 (-0.06, 0.05)
$45,000 to $74,999		0.02 (-0.04, 0.08)		0.02 (-0.04, 0.09)
≥$75,000		**0.07 (0.01, 0.13)**		**0.07 (0.01, 0.13)**
Body mass index (BMI)				
BMI < 25 kg/m^2 [a]				
BMI 25 to 29.9 kg/m^2		0.02 (-0.03, 0.07)		0.03 (-0.02, 0.08)
BMI > 30 kg/m^2		-0.001 (-0.05, 0.05)		-0.002 (-0.05, 0.04)

Values are β coefficient (95% confidence interval)**. Bold type indicates p<0.05**; *italic type indicates p<0.10* for a Wald test comparing the value to the referent or for an increase of one unit. THg = total mercury; NH = Non-Hispanic; BMI = Body mass index. a. Referent

Table S3. Comparison of estimated methylmercury concentrations [a] for selected total mercury concentrations for a fish and shellfish consuming population.

Total mercury, µg/L	Methylmercury, µg/L	
	**Full Adult Population**	**Women of Reproductive Age Population**
0.30	0.18	0.18
0.50	0.36	0.36
0.75	0.58	0.58
1.00	0.80	0.80
1.25	1.05	1.05
1.50	1.31	1.30
1.75	1.56	1.54
2.00	1.81	1.79
2.50	2.32	2.29
3.00	2.82	2.78
**3.40** [b]	**3.22**	**3.18**
3.50	3.33	3.28
4.00	3.83	3.77
4.50	4.34	4.27
5.00	4.84	4.76
**5.80** [b]	**5.65**	**5.55**
6.00	5.85	5.75
10.00	9.89	9.71
15.00	14.94	14.66
20.00	19.99	19.61
25.00	25.04	24.56
30.00	30.09	29.51
35.00	35.14	34.46
40.00	40.19	39.41
45.00	45.24	4.36
50.00	50.29	49.31

a. Uses unadjusted, spline THg model is for calculation. b. The U.S. EPA's current reference dose for MeHg is equivalent to 5.80 µg/L Hg in cord blood; it is also estimated that cord blood Hg is ~1.7 times higher than maternal blood Hg, thus 5.80 µg/L Hg in cord blood is anticipated to be ~3.40 µg/L Hg in maternal blood Hg.

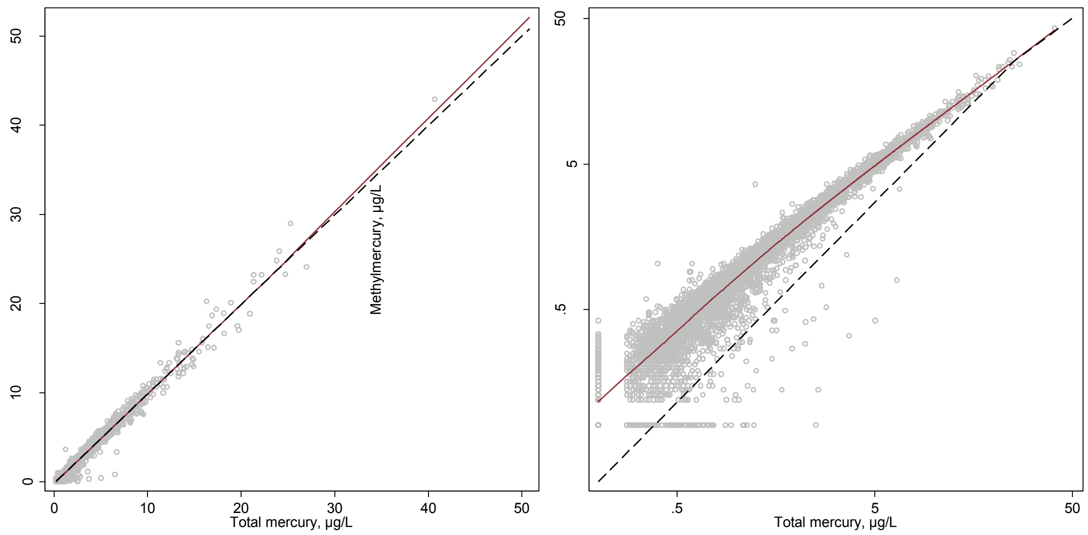

Figure S2: Whole blood total mercury versus whole blood methylmercury among the full adult population from the training dataset, N=5268. The red solid line is a lowess (smoothed) curve; the black dashed line is a linear fitted curve. A: Graph plotted on non-transformed axes. B: The same graph as in A), but plotted on log transformed axes.

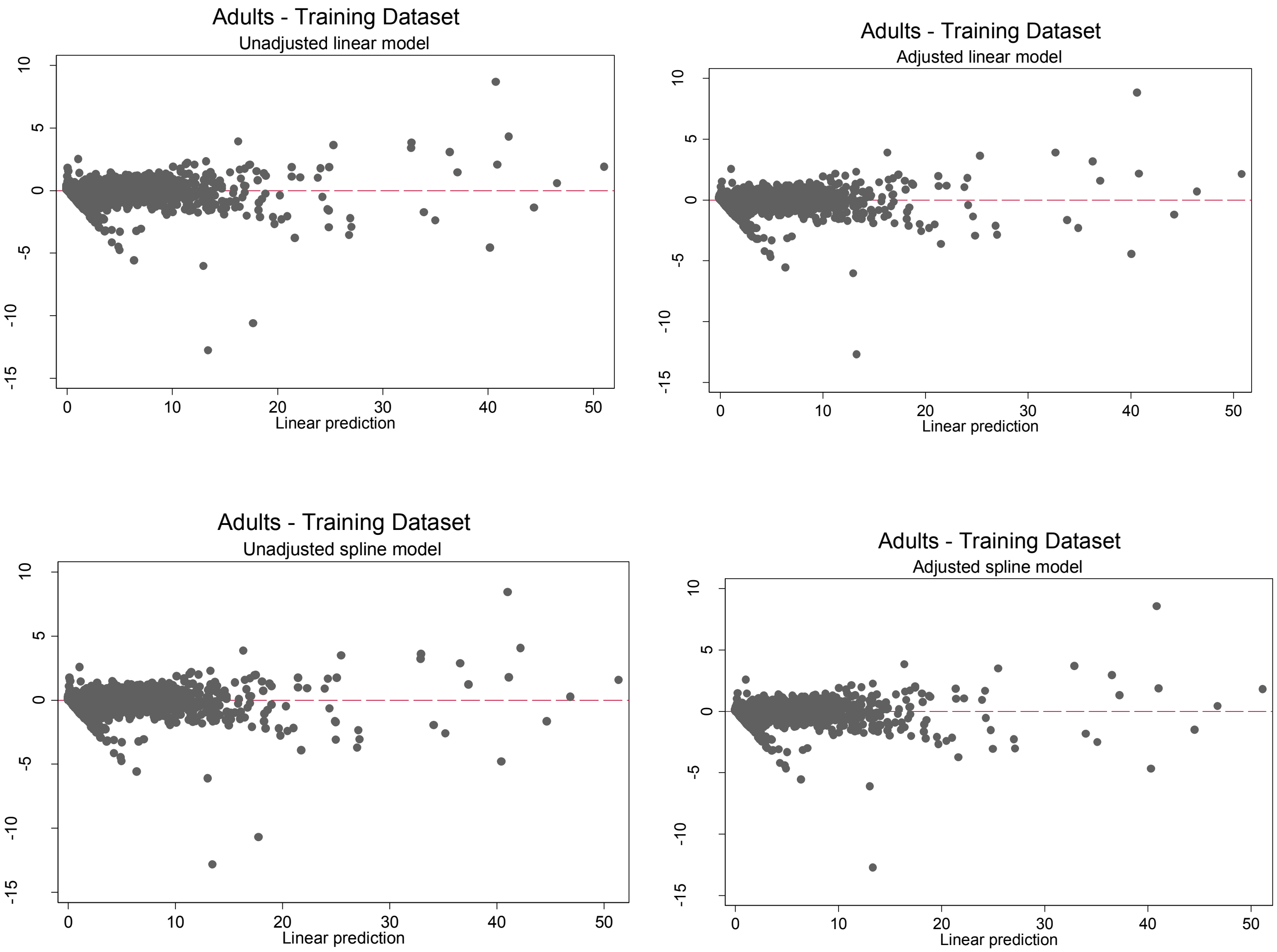

Figure S3: Residual versus fitted plots for methylmercury (MeHg) among fish and shellfish consumers ≥ 15 years old, N=5268, training dataset. Adults = Full Adult Population.

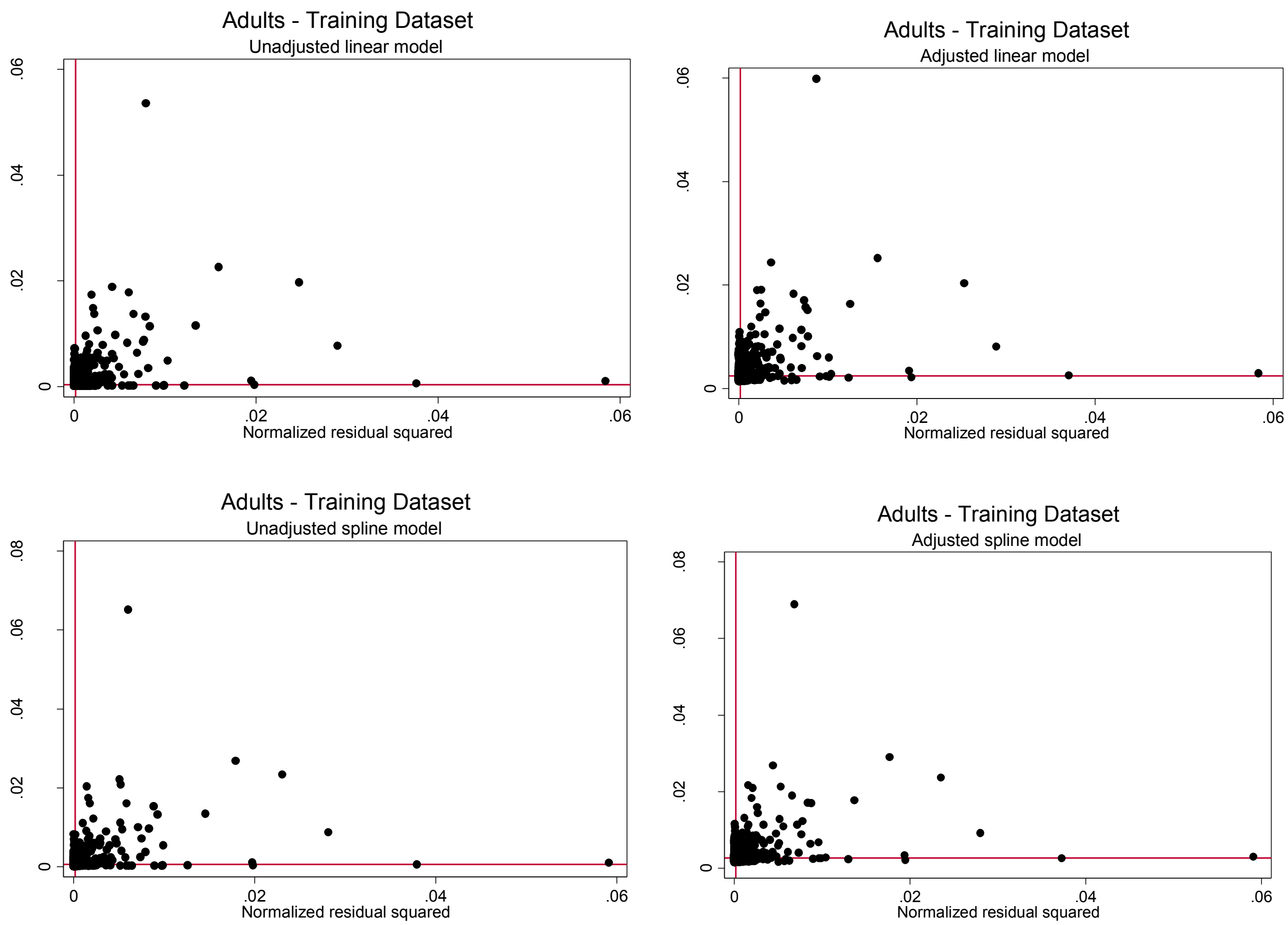

Figure S4: Leverage versus squared residual plots for methylmercury (MeHg) among fish and shellfish consumers ≥ 15 years old, N=5268, training dataset. Adults = Full Adult Population.

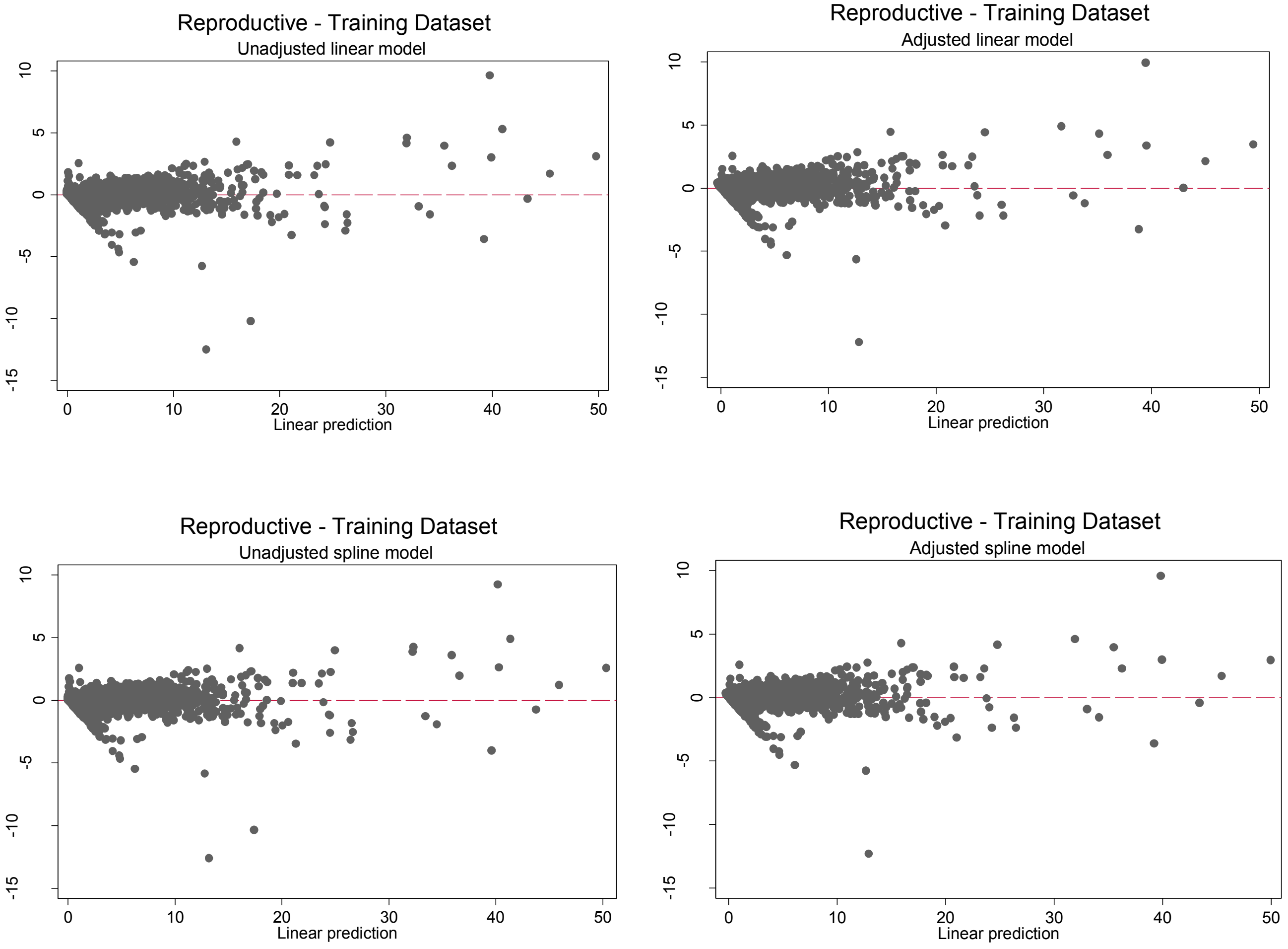

Figure S5: Residual versus fitted plots for methylmercury (MeHg) among female fish and shellfish consumers 15 to 44 years old, N=1285, training dataset. Reproductive = Women of Reproductive Age Population.

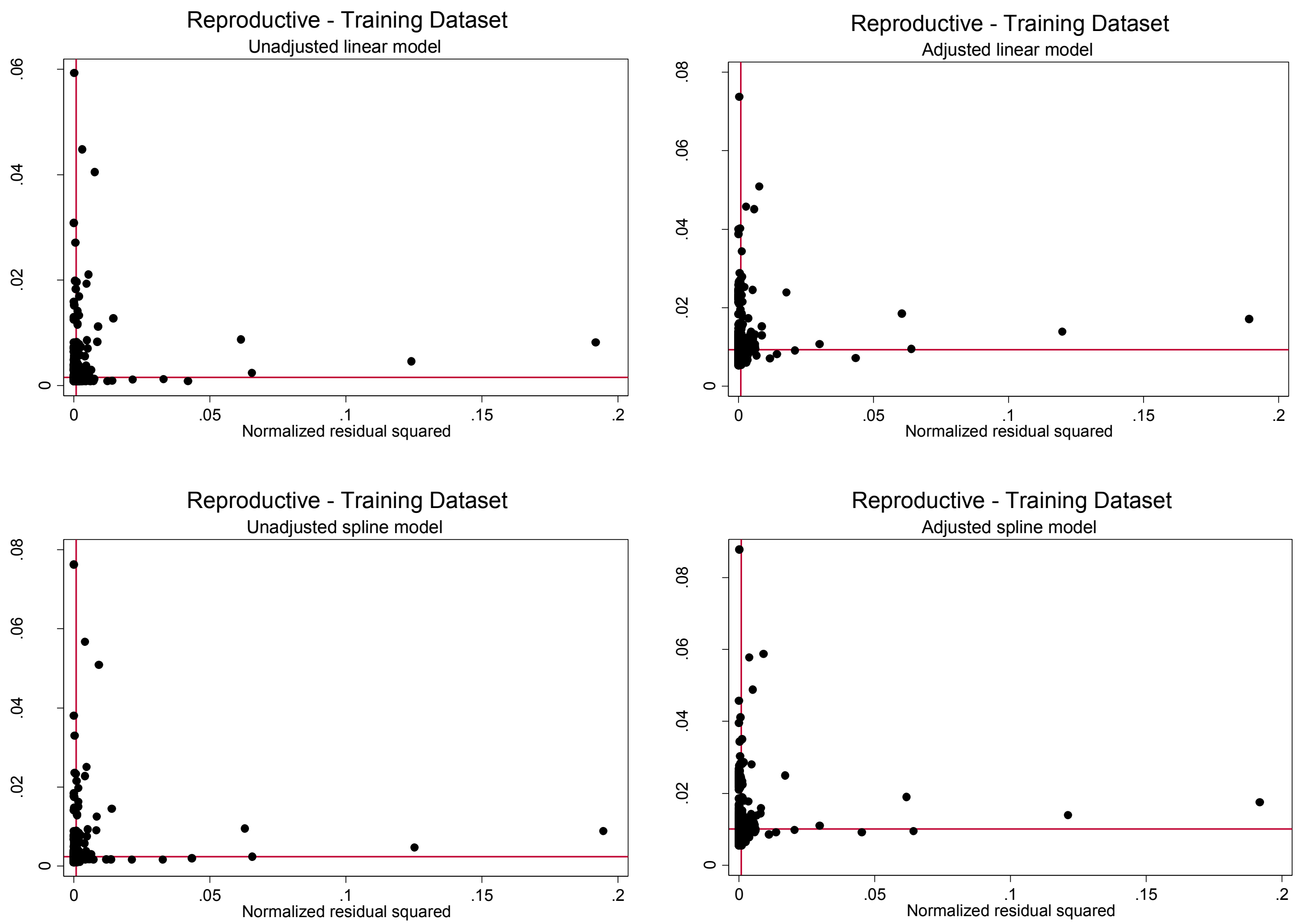

Figure S6: Leverage versus squared residual plots for methylmercury (MeHg) among female fish and shellfish consumers 15 to 44 years old, N=1285, training dataset. Reproductive = Women of Reproductive Age Population.

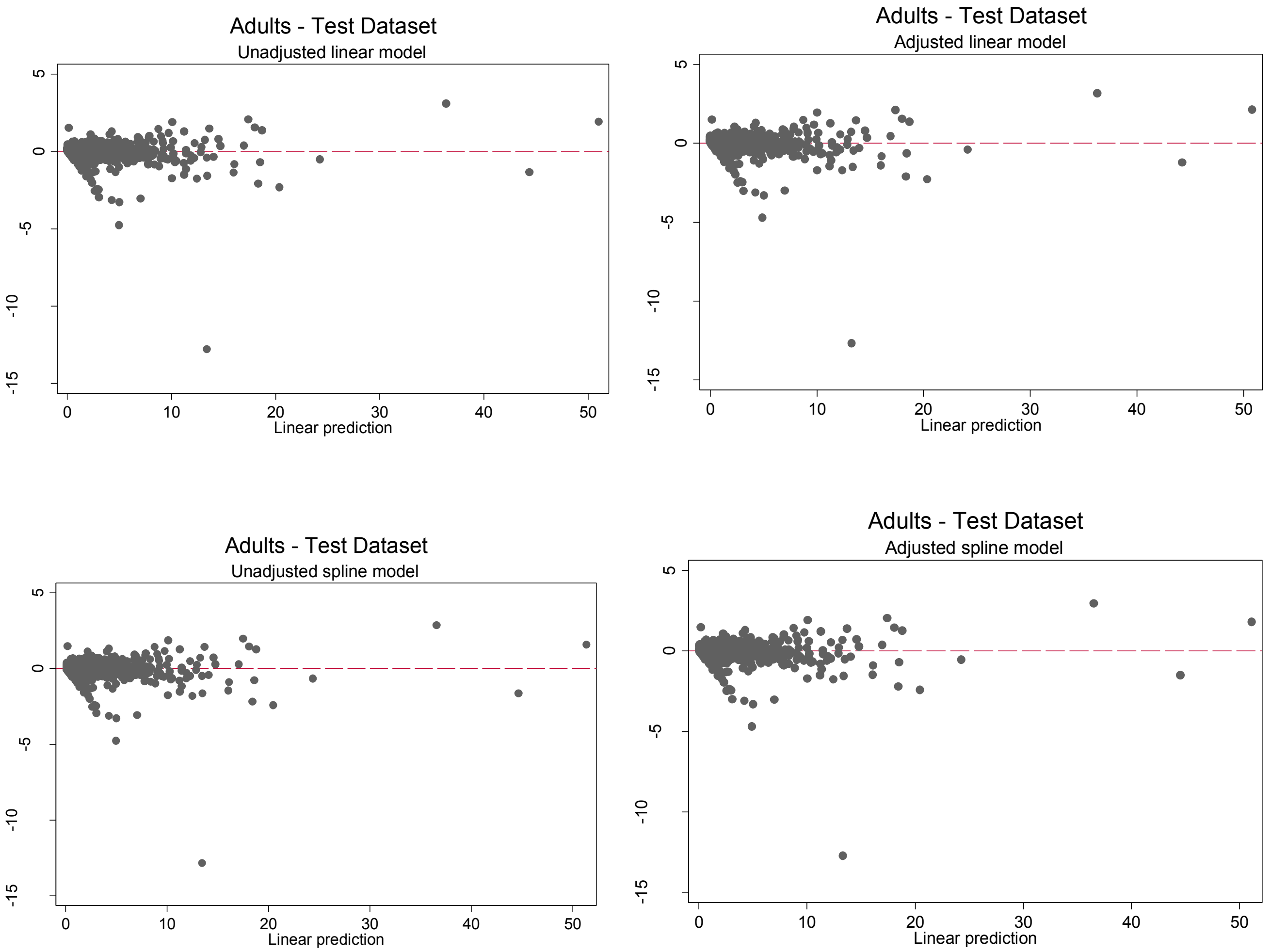

Figure S7: Residual versus fitted plots for methylmercury (MeHg) among fish and shellfish consumers ≥ 15 years old, N=2336, test dataset. Adults = Full Adult Population.

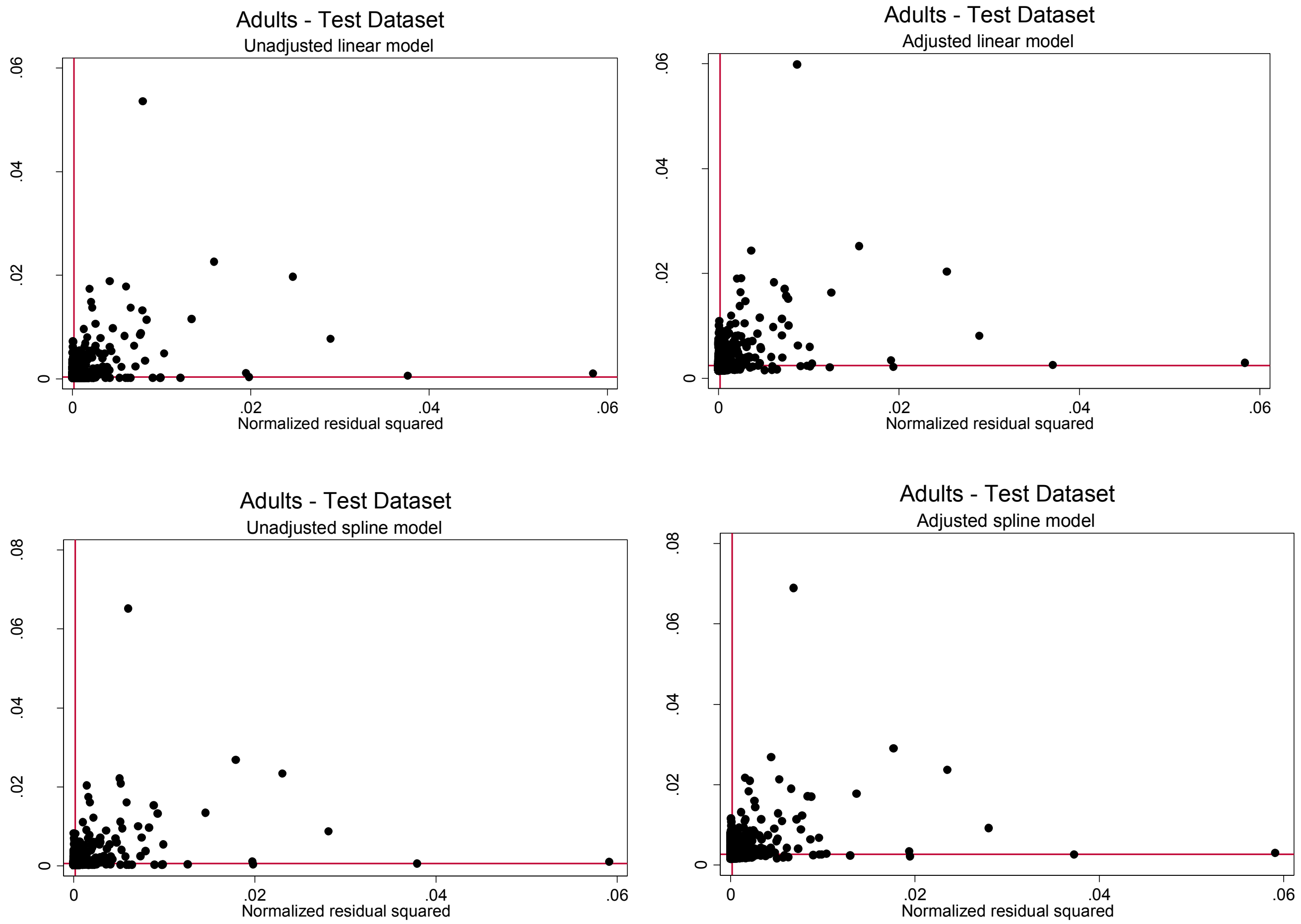

Figure S8: Leverage versus squared residuals plots for methylmercury (MeHg) among fish and shellfish consumers ≥ 15 years old, N=2336, test dataset. Adults = Full Adult Population.

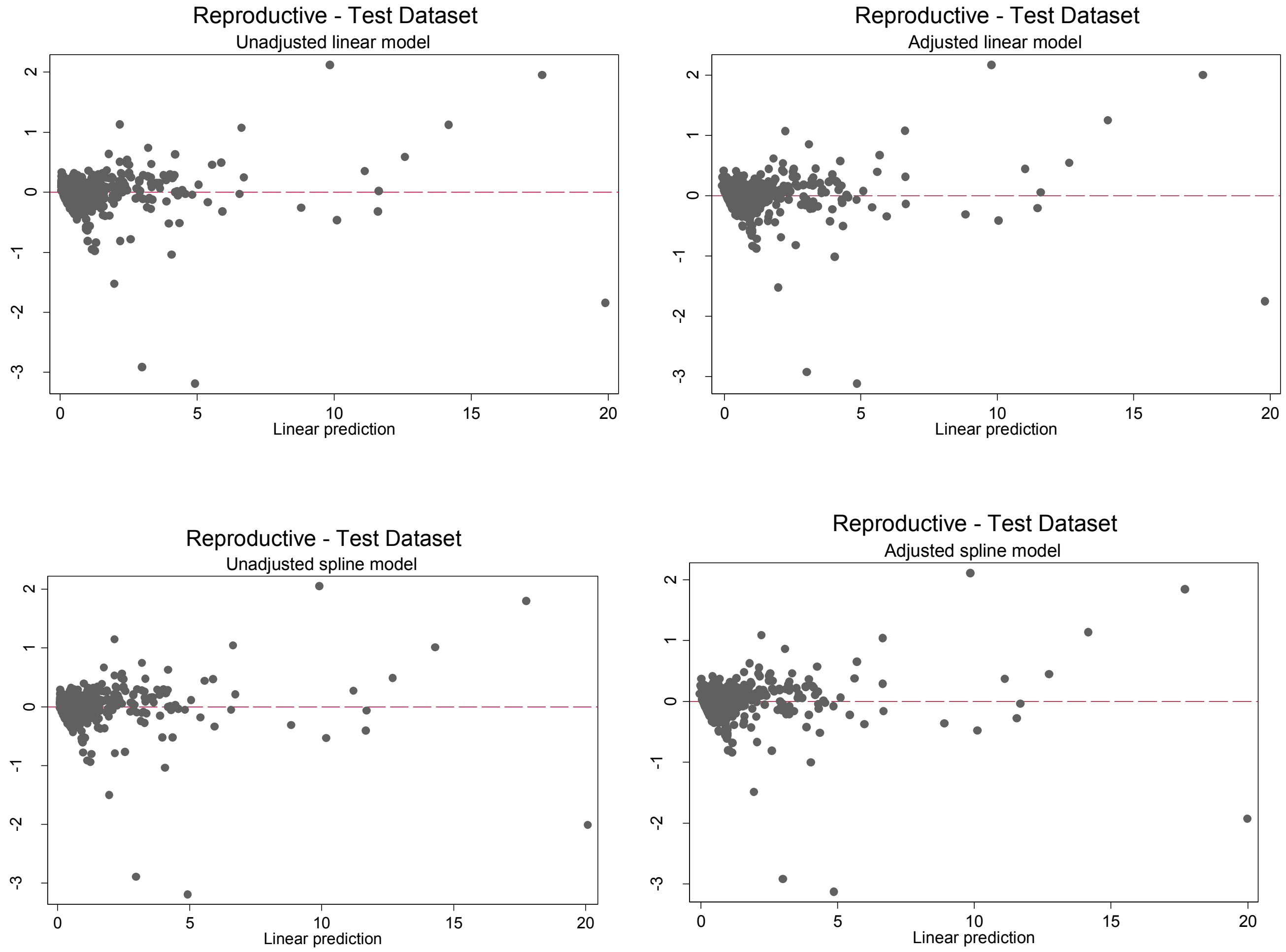

Figure S9: Residual versus fitted plots for methylmercury (MeHg) among female fish and shellfish consumers 15 to 44 years old, N=560, test dataset. Reproductive = Women of Reproductive Age Population.

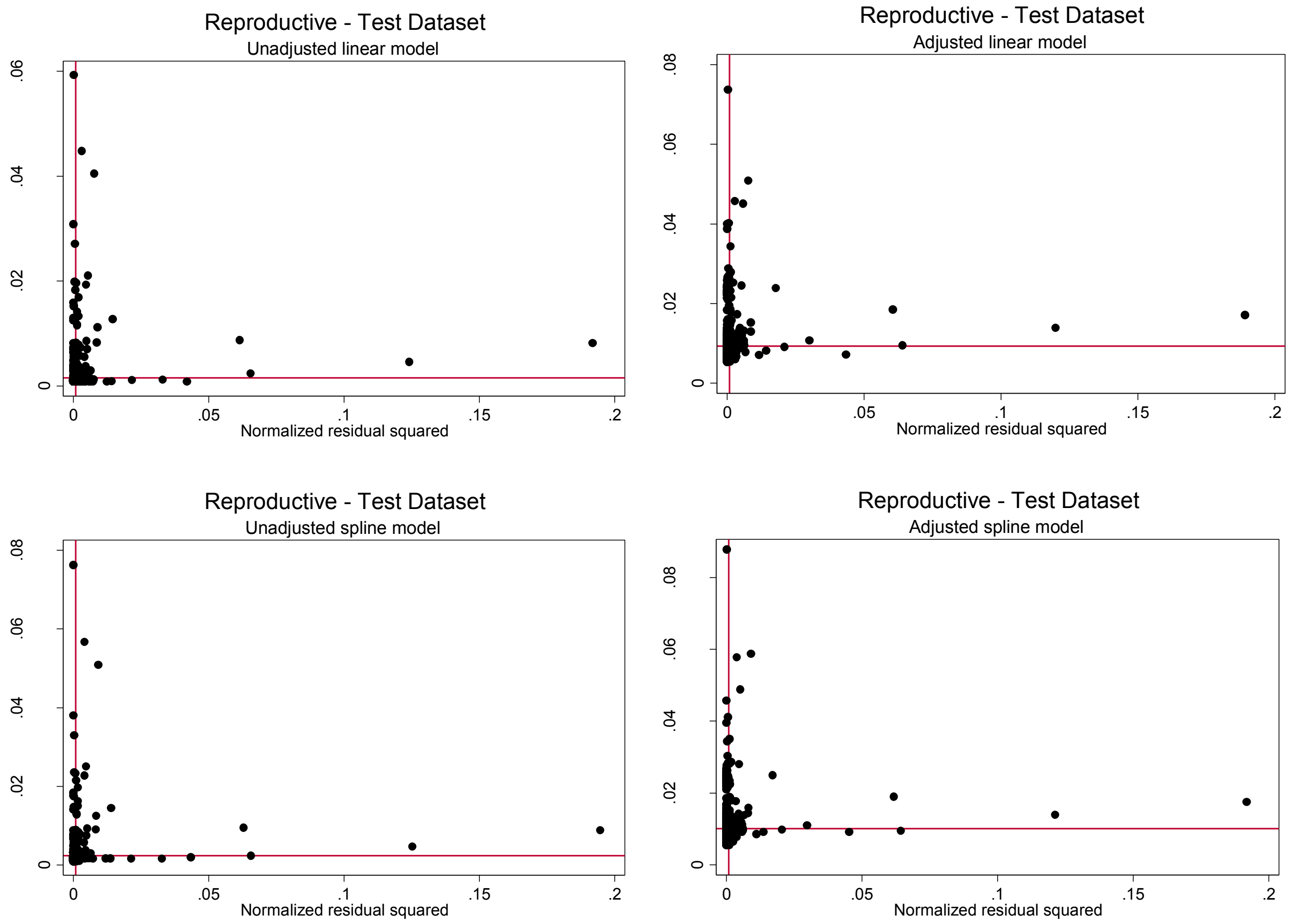

Figure S10: Leverage versus squared residual plots for methylmercury (MeHg) among female fish and shellfish consumers 15 to 44 years old, N=560, test dataset. Reproductive = Women of Reproductive Age Population.
